# Tri et détection du COVID-19 par TDM thoracique low-dose chez des patients tout-venant au service de radiologie de l´hôpital de Fann (Dakar-Sénégal)

**DOI:** 10.11604/pamj.supp.2020.37.1.26140

**Published:** 2020-10-13

**Authors:** Ibrahima Niang, Ibrahima Diallo, Joseph Coumba Ndoffene Diouf, Mamadou Ly, Mouhamadou Hamine Toure, Khadidiatou Ndiaye Diouf, Fallou Galass Niang, Ibrahima Faye, Mama Ndao, Geraud Akpo, Hamidou Deme, Abdoulaye Dione Diop, Sokhna Ba, Elhadji Niang

**Affiliations:** 1 Service d´Imagerie Médicale, Centre Hospitalier National Universitaire de Fann, Dakar, Sénégal,; 2 Service d´Imagerie Médicale, Université Gaston Berger, Saint-Louis, Sénégal,; 3 Service de Pneumologie, Centre Hospitalier National Universitaire de Fann, Dakar, Sénégal,; 4 Service d´Imagerie Médicale, Centre Hospitalier Universitaire Aristide Le Dantec, Dakar, Sénégal

**Keywords:** COVID-19, TDM, low-dose, verre dépoli, PCR, Sénégal, COVID-19, CT scan, ground-glass opacity, PCR, Senegal

## Abstract

**Introduction:**

la COVID-19 s´est rapidement propagée depuis son apparition en Chine devenant actuellement un problème de santé internationale. Son diagnostic définitif se fait par réaction de polymérisation en chaîne (PCR) sur des prélèvements nasopharyngés. Ce moyen diagnostic est de faible sensibilité avec des résultats différés. Ce qui laisse place à la tomodensitométrie thoracique comme moyen diagnostic alternatif. Les objectifs de cette étude étaient d´évaluer la fréquence des lésions TDM évocatrices de COVID-19 et de confronter les résultats de la tomodensitométrie (TDM) à ceux du test PCR.

**Méthodes:**

étude prospective réalisée sur une période de 15 jours ouvrables et qui a porté sur 47 patients. Ces patients étaient recrutés sur la présence d´au moins 2 signes cliniques du COVID-19. Une TDM thoracique sans injection selon le protocole « FAIBLE-DOSE » a été réalisé. Un test PCR sur prélèvements nasopharyngés a été fait chez les patients avec des signes évocateurs de COVID à la TDM. Un test sérologique été réalisé en cas de discordance entre les résultats TDM et PCR.

**Résultats:**

la TDM thoracique était anormale chez 38 patients et normale chez 9 patients. Des lésions évocatrices de COVID-19 ont été identifiées chez 32 patients. Deux patients ont eu des lésions de pneumopathie non spécifique. Des lésions de pneumopathie tuberculeuse ont étés visualisées chez 3 patients. Un patient a eu des lésions de pneumopathie interstitielle commune. La DLP (dose-length product) moyenne était de 59 mGy.cm avec des extrêmes de 25 et 95 mGy.cm. L´opacité en verre dépoli était présente chez 100% des suspects de COVID-19 à la TDM. Le résultat du test PCR a confirmé la TDM chez 12 patients soit une valeur prédictive positive de la TDM de 37,5%. Chez 20 patients avec lésions COVID à la TDM, le test PCR était négatif soit un taux de faux positif de 62,5%. Chez les patients avec test PCR négatif, 4 ont fait un test sérologique de COVID-19 et ce test était positif chez 3.

**Conclusion:**

la TDM thoracique low-dose permet de réduire l´irradiation chez les patients COVID-19 qui sont à risque de dose cumulative par répétition des TDM. La TDM permet d´identifier les lésions évocatrices de COVID-19. Elle permet également le triage des patients en permettant d´identifier d´autres diagnostics.

## Abstract (en)

**Introduction:**

COVID-19 has spread rapidly since its emergence in China and is currently a global health issue. Its definitive diagnosis is made by PCR on nasopharyngeal swabs. However, this diagnostic test has low sensitivity with delayed results. Hence, thoracic computed tomography represents an interesting alternative. The aims of this study were to assess the frequency of computed tomography (CT) lesions suggestive of COVID-19 and to compare the results of CT and PCR test.

**Methods:**

a prospective study carried out over15 working days and involved 47 patients. These patients were recruited based on the presence of at least 2 clinical signs of COVID-19. Chest CT without contrast according to the “LOW-DOSE” protocol was performed. A PCR test on nasopharyngeal swabs was done in patients with signs suggestive of COVID on CT. A serological test was performed in case of a discrepancy between the CT and PCR results.

**Results:**

thoracic CT was abnormal in 38 patients and normal in 9 patients. Lesions suggestive of COVID-19 have been identified in 32 patients. Two patients had lesions of non-specific pneumonia. Tuberculosis lesions were visualized in 3 patients. One patient had lesions of interstitial pneumonia. The mean DLP was 59 mGy.cm with extremes of 25 and 95 mGy.cm. Ground-glass opacity was present in 100% of COVID-19 suspects on CT. The results of the PCR test were the same than CT in 12 patients. The positive predictive value for CT was 37.5%. In 20 patients with COVID lesions on CT, the PCR test was negative with a false positive rate of 62.5%. In the patients with negative PCR test, 4 had a serological test for COVID-19 and this test was positive in 3.

**Conclusion:**

low-dose chest CT can reduce radiation exposure in COVID-19 patients who are at risk of cumulative dose due to repetitive exam. CT can identify lesions suggestive of COVID-19. It also enables the triage of patients by identifying other diagnoses.

## Introduction

Fin décembre 2019, une série de cas de pneumonie provoquée par un nouveau coronavirus est apparue en Chine, et s'est vite répandue sur tous les continents. Cette maladie se transmet d'homme à homme avec une grande contagiosité. Ainsi, elle s´est propagée très rapidement et déclarée «urgence de santé internationale» depuis le 30 janvier 2020 [1]. Au Sénégal, les 2 premiers cas sont détectés le 02 mars 2020 et à ce jour on compte plus de 14500 cas confirmés et 300 décès [2]. Quand la maladie est symptomatique, ses manifestations cliniques sont non spécifiques et comprennent essentiellement la toux et la fièvre associées ou non à la dyspnée, la fatigue, les maux de tête, les douleurs musculaires, la perte du gout et de l´odorat [3,4]. Le diagnostic définitif de l'infection à COVID-19 consiste en la détection de l'ARN viral sur écouvillon nasopharyngé par RT-PCR (Reverse Transcription- Polymerase Chain Reaction) [5]. Les kits de test ne sont pas toujours disponibles et donnent des résultats différés et de sensibilité sub-optimale [6,7]. Cette faible sensibilité fait qu´un test PCR négatif n'exclut donc pas la COVID-19 et plusieurs tests peuvent être nécessaires pour établir le diagnostic final. Ceci peut entraîner plusieurs jours d'incertitude pour les professionnels de santé et d´angoisse pour les patients. A ce jour en l'absence de vaccins et/ou de traitements spécifiques disponibles, il est très important de détecter et d'isoler les patients infectés. Car les patients infectés avec PCR négatif peuvent exposer de nombreuses autres personnes à l´infection s´ils ne sont pas isolés. Dans une revue de l'approche mondiale de la gestion de l'épidémie de COVID-19, les auteurs ont signalé que de nombreux pays utilisent ainsi la tomodensitométrie pour les patients suspects en raison du manque de tests PCR [8]. Une grande étude chinoise, à Wuhan a suggéré l'utilisation de la tomodensitométrie comme méthode de diagnostic alternatif [6]. Le but de ce travail était d´étudier le rôle de la tomodensitométrie low-dose dans le diagnostic de la COVID chez des patients adressés dans un service d´imagerie. Les objectifs spécifiques étaient de: i) Évaluer la fréquence des lésions TDM évocatrices de COVID chez ces patients présentant une suspicion clinique. ii) Confronter les résultats de la TDM à ceux du test PCR sur prélèvements nasopharyngés.

## Méthodes

**Figure 1:**
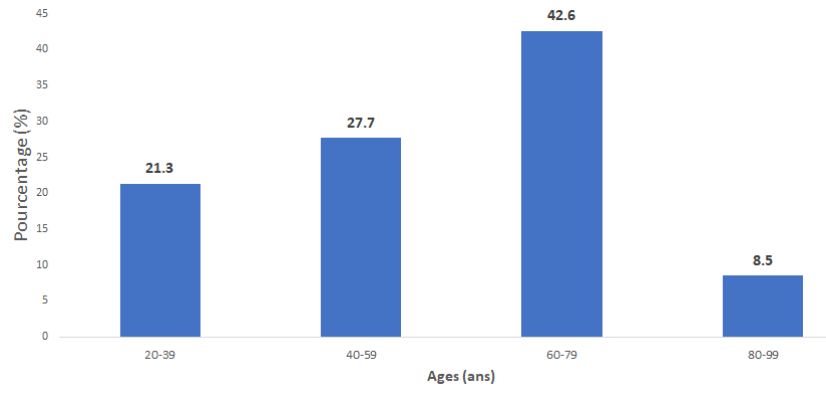
répartition des patients de l´étude selon la moyenne d´âge

Il s´agissait d´une étude prospective réalisée sur une période de quinze (15) jours ouvrables entre le 15 juin et le 3 juillet 2020. Cette étude a été réalisée dans le service d´imagerie médicale du centre hospitalier universitaire national de Fann. L´hôpital de Fann abrite le principal service des maladies infectieuses du pays et le premier centre de traitement épidémiologique du COVID. Le service d´imagerie doté de deux appareils de scanner a ainsi effectué une grande partie des examens tomodensitométriques des patients COVID dans la capitale. Nous avons inclus tout patient reçu au service pour un examen d´imagerie et qui avait au moins 2 signes sur ceux en faveur de la COVID-19, inscrits sur le formulaire de tri COVID. Ces signes étaient: fièvre > = 38°C, céphalées, dyspnée, toux, anosmie, agueusie, mal de gorge, écoulement nasal. Nous avons exclu les patients adressés au service explicitement pour suspicion de COVID-19 ou non consentant à la réalisation de la TDM et/ou du test PCR. Ainsi nous avions colligé au total 47 patients. Cette population d´étude était composée de 28 femmes (59,6%) et de 19 hommes (40,4 %) soit un sex-ratio de 0,68. L´âge moyen de notre série était de 57 ans avec un écart-type de 17,7 ans et des extrêmes de 22 et 92 ans. La tranche d´âge [60- 79 ans] était la plus représentée avec 42,6%. La répartition des patients selon les tranches d´âge est représentée par la Figure 1. Les examens TDM ont été réalisés sur un appareil HITACHI® SCENARIA de 64 barrettes, selon un protocole « FAIBLE-DOSE » préréglé par la machine et sans injection de produit de contraste. Les tests PCR ont été réalisés sur des prélèvements nasopharyngés chez les patients qui avaient des lésions TDM évocatrices de COVID-19. Les tests sérologiques ont été réalisés sur prélèvements sanguins chez les patients avec discordance TDM-PCR. Nous avons recherché les différents patterns radiologiques et leur topographie, en faveur du diagnostic de COVID-19 ou d´un autre diagnostic différentiel: 1) Verre dépoli: zone de parenchyme pulmonaire dont la densité est augmentée, sans pour autant effacer les vaisseaux pulmonaires. 2) Crazy-paving: verre dépoli associe à des réticulations septales, 3) Condensation: augmentation systématisée de la densité pulmonaire effaçant les vaisseaux pulmonaires. 4) Micronodules: nodule de petite taille (moins de 10 mm) localisé dans une zone pulmonaire L´analyse des données a été effectuée avec le logiciel SPSS (Statistical package for Social Sciences) version 18.

## Résultats

**Figure 2:**
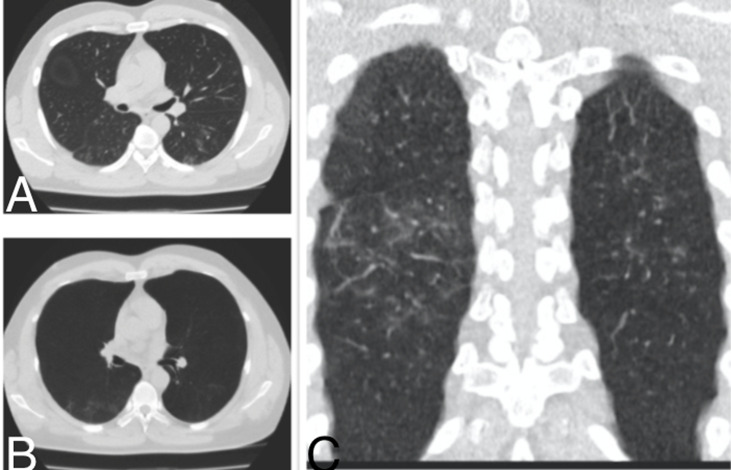
pneumopathie COVID d´étendue minime d’un patient de 55 ans; TDM thoracique en fenêtre pulmonaire: A) coupe axiale, B) coupe axiale en MinIP, C) reconstruction coronale: discrètes plages d´opacité en verre dépoli sous pleurales bilatérales au niveau des parties postéro-moyennes des poumons avec prédominance à droite

**Figure 3:**
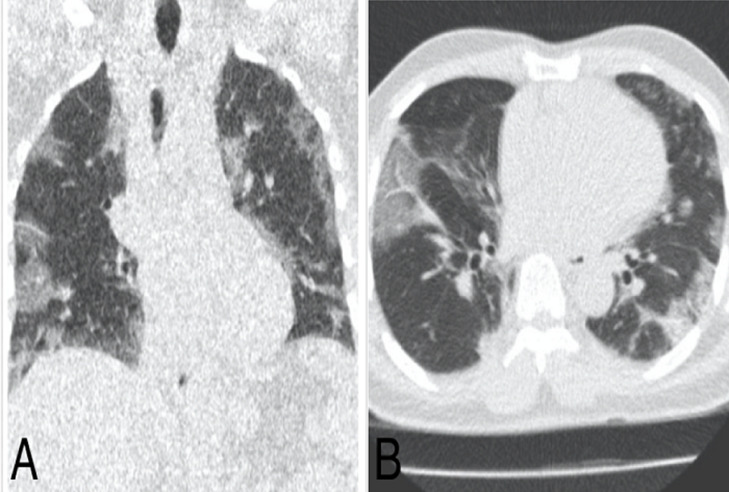
pneumopathie COVID d´étendue modérée d’un patient de 62 ans; TDM thoracique en fenêtre pulmonaire: A) reconstruction coronale, B) coupe axiale: on note plusieurs plages de verre dépoli sous pleurales bilatérales, étendues en hauteur et prédominant aux bases

**Figure 4:**
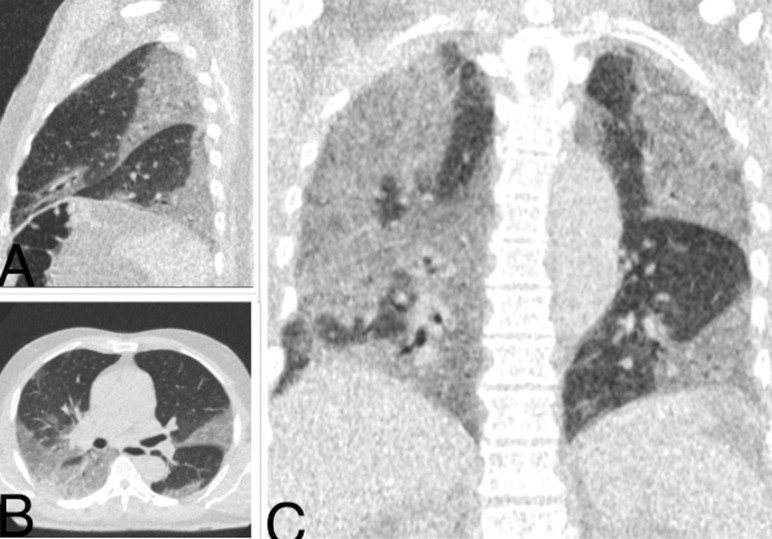
pneumopathie COVID d´étendue sévère d’un patient de 71 ans; TDM thoracique en fenêtre pulmonaire: A) reconstruction sagittale, B) coupe axiale, C) reconstruction coronale: on note multiples plages de verre dépoli sous pleurales et centrales bilatérales, laissant peu de zones saines

**Figure 5:**
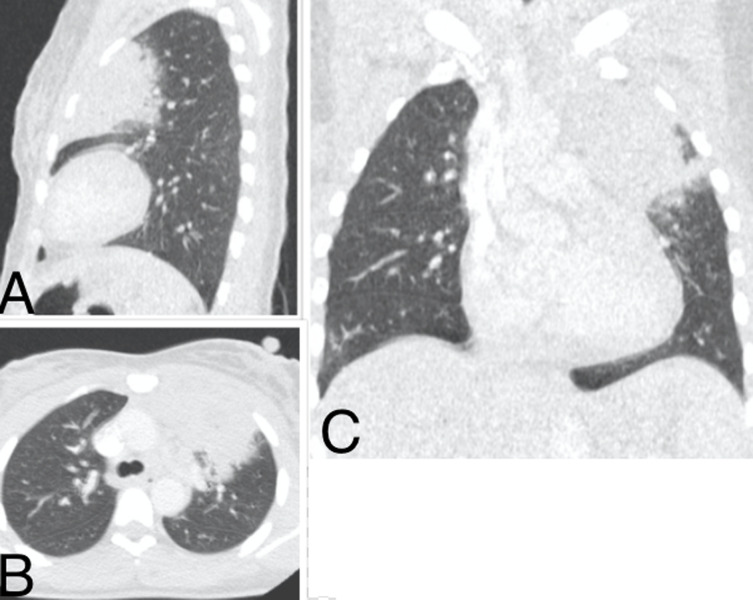
pneumopathie non spécifique d’une patiente de 37 ans; TDM thoracique en fenêtre pulmonaire: A) reconstruction sagittale, B) coupe axiale, C) reconstruction coronale: on note une condensation pulmonaire du segment antérieur du lobe supérieur gauche (S3G)

**Figure 6:**
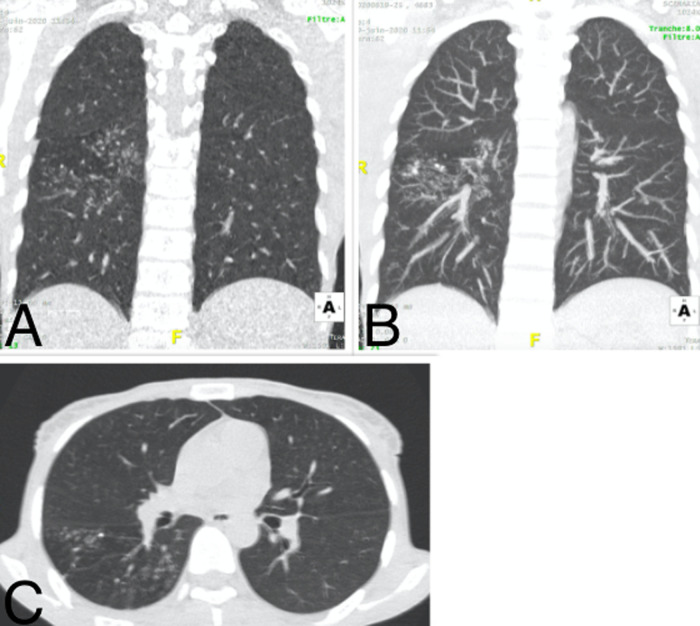
pneumopathie tuberculeuse d’une patiente de 65 ans; TDM thoracique en fenêtre pulmonaire: A) reconstruction coronale, B) reconstruction coronale en MIP, C) coupe axiale: foyer de micronodules branchés donnant l´aspect « d´arbre en bourgeon » au niveau des segments S9 et S10 droits; il s´y associe des nodules calcifiés adjacent

**Figure 7:**
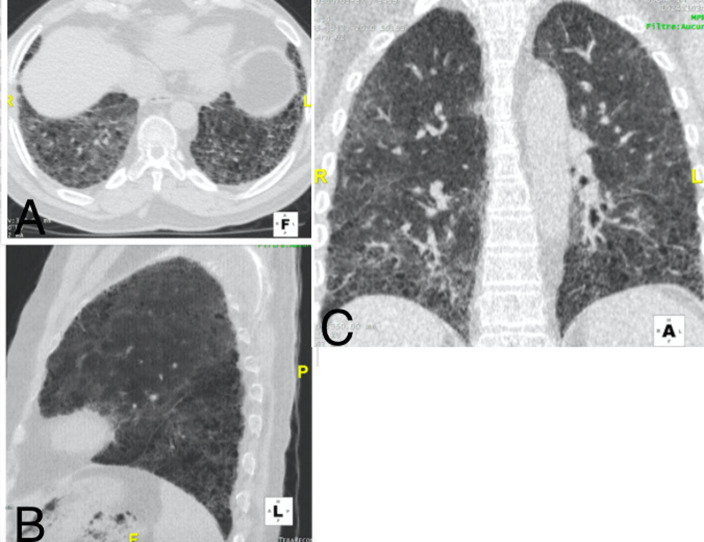
pneumopathie interstitielle commune (PIC) d’un patient de 48 ans; TDM thoracique en fenêtre pulmonaire: A) coupe axiale, B) reconstruction sagittale en MinIP, C) reconstruction coronale: opacité en verre dépoli diffus associé à un aspect de « rayon de miel » sous pleural bilatéral à prédominance basale

L´examen était anormal chez 38 patients (80,9%) et normal chez 9 patients (19,1%). Des lésions évocatrices de COVID-19 ont été identifiées chez 32 patients soit 68,1% [Figure 2 (pneumopathie COVID d´étendue minime, patient de 55 ans, initialement envoyé au service d´imagerie pour une échographie abdominale, avec 3 signes sur la fiche de tri: dyspnée, fatigue et céphalée); Figure 3 (pneumopathie COVID d´étendue modérée: patient de 62 ans, initialement envoyé au service d´imagerie pour une radiographie thoracique, il avait 2 signes sur la fiche de tri: dyspnée, fatigue); Figure 4 (pneumopathie COVID d´étendue sévère, patient de 71 ans, initialement envoyé au service d´imagerie pour une radiographie thoracique avec 2 signes sur la fiche de tri: dyspnée, toux)]. Deux patients soit 4,3% ont eu des lésions de pneumopathie non spécifique (Figure 5). Des lésions de pneumopathie tuberculeuse ont étés visualisées chez 3 patients soit 6,4% (Figure 6). Un patient a eu des lésions de pneumopathie interstitielle commune (Figure 7). La DLP moyenne était de 59 mGy.cm avec des extrêmes de 25 et 95 mGy.cm. L´opacité en verre dépoli était présente chez tous les patients suspects de COVID à la TDM. Ce verre dépoli était uniquement sous pleural chez 81% des patients et mixte (central et périphérique) chez le reste. L´aspect de crazy-paving y était associé chez un patient. Une condensation était également associée chez un patient. Un (1) patient avait des lésions de verre dépoli associé à des kystes sous pleuraux avec aspect de « rayon de miel » en faveur d´une PIC. Deux (2) patients avaient des condensations pulmonaires lobaires systématisées faisant évoquer en premier lieu une pneumopathie non spécifique. Trois (3) patients avaient des micronodules branchés avec aspect « d´arbre en bourgeons » plus ou moins associés à des condensations, faisant évoquer une pneumopathie tuberculeuse. Les lésions évocatrices de COVID d´étendue modérée était plus fréquente avec 50% et il n´y avait de lésions COVID d´étendue critique parmi nos patients (Table 1). Le résultat du test PCR a confirmé les résultats tomodensitométriques chez 12 patients soit une valeur prédictive positive (VPP) de la TDM de 37,5%. Chez 20 patients avec lésions COVID à la TDM, le test PCR était négatif soit un taux de faux positif de 62,5%. Chez les 20 patients avec test PCR négatif, 4 ont accepté de revenir pour faire un test sérologique de COVID-19 et le test sérologique a été positif chez 3 d´entre eux.

**Tableau 1:** répartition des lésions COVID en fonction de l’étendue sur le parenchyme

Etendue des lésions COVID	Effectifs	%
COVID MINIME (< 10 %)	6	18,8
COVID MODEREE (10-25 %)	16	50,0
COVID ETENDUE (25-50 %)	8	25,0
COVID SEVERE (50-75 %)	2	6,3
COVID CRITIQUE (>75 %)	0	0
**Total**	32	100,0

## Discussion

L´âge des patients avec des lésions suspectes de COVID à la TDM allait de 27 à 92 ans et celui des patients avec PCR positifs de 35 à 92 ans, ce qui montre un large éventail d´âge aussi bien chez les patients suspectés par la TDM que confirmés par le test PCR. Néanmoins la tranche d´âge 60-80 ans était celle avec le plus de lésions suspectes de COVID, ce qui reflète les données épidémiologiques des patients atteints de COVID [3]. La moyenne des DLP chez nos patients (59 mGy.cm) était 5 fois moins élevée que les niveaux de références diagnostiques européennes pour une TDM thoracique (348 mGy.cm) [9]. Les demandes de TDM thoraciques ont largement augmenté durant cette période de pandémie à infection respiratoire et certains patients pourraient avoir à faire plusieurs TDM sur une courte période [10]. L'effet cumulatif de ces examens répétés pouvant augmenter considérablement la dose d´irradiation de ces patients. En prenant en compte le principe de ALARA (As Low As Reasonably Achievable) toute TDM réalisée devrait l´être sur la base d´arguments cliniques et avec une bonne optimisation des doses d´irradiation [11]. La TDM low-dose à la place du protocole classique s´impose pour limiter cette irradiation. La tomodensitométrie était normale chez 9 patients. Ces patients n´ont pas eu de test PCR par la suite bien qu´une TDM normale n´exclut pas le diagnostic de COVID. D´autant plus qu´on sait que les lésions de COVID à la TDM peuvent être absentes dans les 48 premières heures de l´infection [12].

Des lésions suspectes de COVID ont étés visualisées chez 68,1% des patients et toutes ces patients avaient des lésions de verre dépoli avec une distribution sous pleurales prédominantes. L´étude de Guan *et al*. [13] sur 57 patients retrouvait une même proportion de lésions COVID à la TDM avec 88,7% et également la présence de verre dépoli chez tous ces patients. Ceci démontre la sensibilité du verre dépoli et surtout de sa distribution sous pleurale pour le diagnostic de COVID-19 malgré le fait qu´il ne soit spécifique d´aucune pathologie. Néanmoins il devrait être analysé avec prudence et l´examen réalisé en inspiration profonde pour éviter les surdiagnostics de COVID à la TDM par faux verre-dépoli [14]. Un seul patient présentait des lésions de crazy-paving associé au verre dépoli et également un seul patient avait une condensation associée chez les patients suspects de COVID-19. La faible présence de ces deux lésions chez nos patients s´explique par le fait que ces lésions se retrouvent plus tardivement dans l´évolution de la COVID-19 ou en cas de surinfection pour ce qui est de la condensation pulmonaire [15,16]. Nos patients étaient essentiellement diagnostiqués à une phase plus précoce où on ne retrouve que du verre dépoli. Le seul patient qui avait du verre dépoli, sans pour autant être étiqueté COVID avait des lésions typiques d´une pneumopathie interstitielle commune (PIC). Ces pneumopathies interstitielles diffuses constituent des diagnostics différentiels de la COVID-19 à la TDM. Les autres diagnostics différentiels du COVID retrouvés chez nos patients étaient la tuberculose et la pneumopathie non spécifique. Cette possibilité d´exclure des lésions COVID et de proposer des diagnostics alternatifs constituent l´un des atouts majeurs de la TDM. Et ceci est d´autant plus important en cas de forte affluence de patients où le tri des patients COVID et non COVID devient primordial pour limiter la propagation de l´infection. L´autre avantage de la TDM est la courte durée d´acquisition et la disponibilité immédiate des résultats qui facilitent également le tri des patients et diminue ainsi le risque de propagation du virus.

Les résultats du test PCR ont confirmé ceux de la TDM chez 12 patients donnant à la TDM une valeur prédictive positive (VPP) de 37, 5%. Cette valeur est faible comparée à d´autres études semblables comme celle de Guan *et al*. [13] qui trouvait une VPP de 91,1%. Cette différence sur la VPP pourrait s´expliquer par des faux-négatifs des tests PCR qui sont fréquents, en rapport avec leur faible sensibilité [7,17]. Les arguments diagnostics à la TDM ont été les mêmes aussi bien chez les 12 patients confirmés par les tests PCR que chez les 20 patients non confirmés. Ce qui nous amène à dire que soit ces patients n´étaient plus porteur du virus ou d´une quantité détectable du virus au moment du test PCR soit le test PCR n´était pas fiable. D´ailleurs 3 patients qui avaient des PCR négatifs ont eu une sérologie COVID positive (IgG +) par la suite prouvant ainsi qu´ils étaient infectés sans pour autant que le test PCR ne le démontre. C´est pourquoi la répétition des tests PCR est recommandé chez les patients lorsqu´il y a une suspicion clinique associée à des signes tomodensitométriques de COVID [18]. L´autre explication à la faible valeur de VPP pourrait être le fait que dans notre étude seuls les patients avec des signes tomodensitométriques évocateurs de COVID ont été testés. Le défaut de tests chez tous les patients constitue une limite à cette étude car rend impossible le calcul de la valeur prédictive négative, de la sensibilité et de la spécificité de la TDM. Mais cette indisponibilité des tests PCR est également une raison de plus pour recourir à la TDM pour une prise en charge rapide et efficace surtout en cas de forte affluence de patients symptomatiques.

## Conclusion

La gestion de la COVID-19 est actuellement un problème de santé publique internationale. L´expansion rapide du virus et l´augmentation du nombre de décès de jour en jour doivent amener à un diagnostic alternatif autre que le PCR. La TDM low-dose permet de limiter les doses d´irradiation tout en fournissant des images de qualité. Ce qui laisse la possibilité d'utiliser ses résultats comme aide au diagnostic et comme outil de triage chez les patients suspects et à défaut de trouver des lésions évocatrices de COVID, la TDM peut permettre d'identifier d'autres diagnostics différentiels.

### Etat des connaissances sur le sujet

- Le test PCR est de faible sensibilité dans le diagnostic de la COVID-19;
- La TDM thoracique peut montrer des signes évocateurs de la COVID.

### Contribution de notre étude à la connaissance

- La TDM avec un protocole low-dose diminue considérablement l’irradiation avec une qualité d’image préservée permettant d’identifier les éléments évocateurs de la COVID-19 ou d’un autre diagnostic alternatif;
- En cas de discordance entre le test PCR et les résultats de la TDM thoracique, le test sérologique pourrait aider à départager.
